# A pilot study to investigate the risk of SARS-CoV-2 household transmission following hospital discharge of confirmed cases in Vientiane Capital, Lao PDR

**Published:** 2021-12

**Authors:** Mayfong Mayxay, Phimpha Paboriboune, Xaipasong Xaiyaphet, Khamfong Kunlaya, Manivanh Vongsouvath, Bountoy Sibounheuang, Audrey Dubot-Pérès, Andrew J. H. Simpson, Matthew T. Robinson, Khamla Choumlivong, Vangnakhone Dittaphong, Manichanh Thongsana, Anouphet Chanthamavong, Phetkim Sayasene, Bandith Soumphonphakdy, Khamphoua Soutthisombat, Bouathep Phoumindr, Rattanaxay Phetsouvanh, Phonepadith Xangsayarath, Elizabeth A Ashley

**Affiliations:** 1 Lao-Oxford-Mahosot Hospital-Wellcome Trust Research Unit (LOMWRU), Microbiology Laboratory, Mahosot Hospital, Vientiane Capital, Lao PDR; 2 Institute of Research and Education Development (IRED), University of Health Sciences, Ministry of Health, Vientiane Lao PDR; 3 Centre for Tropical Medicine and Global Health, Nuffield Department of Medicine, University of Oxford, Oxford, UK; 4 Centre d'Infectiologie Lao-Christophe Mérieux (CILM), Ministry of Health, Lao PDR; 5 Unité des Virus Émergents (UVE: Aix-Marseille Univ-IRD 190-Inserm 1207), Marseille, France; 6 Setthathirat Hospital, Vientiane capital, Lao PDR; 7 Mittaphab Hospital, Vientiane capital, Lao PDR; 8 Children Hospital, Vientiane capital, Lao PDR; 9 Department of Health Care and Rehabilitation, Ministry of Health, Lao PDR; 10 Department of Communicable Disease Control, Ministry of Health, Lao PDR; 11 National Center for Laboratory Epidemiology, Ministry of Health, Lao PDR

**Keywords:** SARS-CoV-2, Hospital discharge, Household Risk, Lao PDR

## Abstract

**Background:**

Global guidelines from the World Health Organization on discharging patients diagnosed with COVID-19 changed in 2021 to a symptom-based rather than negative PCR-based approach. Studies have shown that shedding of viable virus continues for approximately eight days after symptom onset in most patients. In Vientiane, Laos, until now, patients diagnosed with asymptomatic or mild COVID-19 are hospitalised for 2 weeks and then, if they still test PCR positive for SARS-CoV-2, stay for a further week in a designated quarantine hotel before being discharged home.

**Objective:**

The aim of this pilot study was to investigate the risk of transmission of SARS-CoV-2 to household contacts of discharged patients who are still PCR-positive following 2-3 weeks quarantine in Vientiane, Lao PDR.

**Methods:**

Adult participants, who were resident in Vientiane Capital and who were about to be discharged from hospital (after 2 weeks hospitalisation), or from a quarantine hotel, following a further one-week quarantine, were screened to assess eligibility for the study. The household of each case was visited a maximum of 48 hours before or up to 24 hours after the participant was discharged and a nasopharyngeal swab was taken from all household members. Repeat nasopharyngeal swabs from cases and contacts were taken on day 7 and day 14 after discharge home of each case.

**Results:**

Between 20th May 2021 and 27th August 2021, 55 cases and 84 contacts in 27 households were enrolled in the study. The median [range] age of all 139 included participants was 26.5 years [3 months to 83 years] and 83 (60%) were female. By household, the median [range] number of cases and contacts were 1 [1-6] and 3 [1-13] respectively. At discharge home 32/48 (67%) cases tested positive for SARS-CoV-2. By day 7 11 of 47 cases (23%) still tested positive for SARS-CoV-2 by PCR and by day 14 this number was 2/24 (8%). No contacts tested positive during follow up and the numbers tested at the time of discharge of the case, 7 days later and 2 weeks later were 56, 57 and 37 respectively. Loss to follow up at day 7 and day 14 ranged from 15-50% (participants not at home at the time of visits).

**Conclusion:**

In this pilot study we found no evidence of onward transmission of SARS-CoV-2 to contacts of cases discharged home with a positive PCR result. This suggests the current discharge policy for mild to moderate COVID-19 case following 2 weeks in hospital in the Lao PDR is safe.

## Introduction

In December 2019 a large number of cases of a new severe acute respiratory syndrome was documented in Wuhan, China, now known as Coronavirus Disease 2019 (COVID-19). The cause was subsequently identified as a novel coronavirus named severe acute respiratory syndrome coronavirus 2 (SARS-CoV-2).^1,2^ A global pandemic was declared on March 12^th^, 2020. The first cases in Laos were diagnosed on March 23^rd^ 2020. Following an early national lockdown and with the introduction of strict border controls and screening, Laos successfully avoided widespread community transmission of SARS-CoV-2 until April 2021. Unfortunately, the situation changed in April when importation of cases infected by the more transmissible B 1.1.7 variant from neighbouring countries, who bypassed border controls, was followed by rapid spread to a number of individuals. The first cases of the delta variant of SARS-CoV-2 were confirmed in July 2021 and this is now the dominant variant in the country.

The current policy in Laos is to admit all confirmed cases of COVID-19 to a healthcare facility. Early in the pandemic patients were discharged once two consecutive nasopharyngeal swabs tested negative for SARS-CoV-2 by PCR, regardless of symptoms. It is now well described that SARS-CoV-2 RNA shedding may be prolonged (2 months or more) in individuals infected with SARS-CoV-2.^3–6^ However there is also evidence that transmission is most likely to occur in the pre-symptomatic and early symptomatic period.^7^ In a study of 100 cases in Taiwan who were not hospitalised and self-isolated at home, the attack rate among household contacts was 4.6% [95% CI, 2.3%-9.3%]. Virus isolation experiments using cell culture in one set of 90 RT-PCR (reverse transcriptase-polymerase chain reaction) SARS-CoV-2-positive samples in a study in Canada showed no growth in samples with cycle threshold (Ct) >24 or more than 8 days after symptom onset.^8^ A similar study from the Netherlands found the median duration of shedding infectious virus was 8 days post onset of symptoms (IQR 5–11). Viral loads above 7 log_10_ RNA copies/mL (odds ratio [OR] of 14.7 (CI 3.57-58.1; *p* < 0.001) were associated with isolation of infectious virus.^6^ Other groups have demonstrated replicating virus as late as 18 days after symptom onset in rare cases.^5^ Even later (months) shedding of infectious virus has been reported from severely immunocompromised patients.^9^

In a systematic review and meta-analysis the mean duration of SARS-CoV-2 RNA shedding was 17.0 days (95% CI 1.·5–18.6; 43 studies, 3229 individuals) in the upper respiratory tract, 14.6 days (9.3–20.0; seven studies, 260 individuals) in lower respiratory tract, and 17.2 days (14.4–20.1; 13 studies, 586 individuals) in stool.^10^ The maximum duration of SARS-CoV-2 shedding in the upper respiratory tract was 83 days and in stool was 126 days. Duration of shedding was associated with age. Importantly, in the studies included in this analysis, viable virus was not detected later than nine days after the onset of symptoms.

The World Health Organization and many countries have changed their discharge guidance from a test-based approach to a symptom-based approach, with special consideration for patients who are immunocompromised.^11^ WHO guidance recommends the following criteria for discharging patients from isolation without retesting:

“*For symptomatic patients: 10 days after symptom onset, plus at least 3 additional days without symptoms*

*For asymptomatic cases: 10 days after positive test for SARS-CoV-2”*

Laos adopted this guidance in May 2021 with a modification to enforce isolation for at least 14 days for asymptomatic individuals in line with their quarantine policy for travellers entering the country), and 17 days for symptomatic patients.However, there was still concern about the safety of this since Laos was still pursuing an aggressive policy of containment, and patients in Vientiane capital have been spending a further 7 days in quarantine in designated hotels. In this pilot study we investigated the risk of transmission to household contacts of discharged patients.

The primary objective of this prospective observational pilot study was to investigate whether household transmission of SARS-CoV-2 occurs within 14 days of discharge from hospital or quarantine facilities.

## Methods

Adult participants, who were resident in Vientiane Capital and who were about to be discharged from a hospital or quarantine hotel following 2-4 weeks’ quarantine and who still tested positive by RT-PCR (Centre d'Infectiologie Lao-Christophe Mérieux, CILM) for SARS-CoV-2 from a nasopharyngeal swab taken in the 48 hours before discharge were screened to assess eligibility for the study. Only consenting participants living in a house or apartment with other family members were included. Residence in student dormitory, shared accommodation for >10 workers, and prisons were all exclusion criteria. The household of each enrolled index case was visited a maximum of 48 hours before or up to 24 hours after the participant was discharged. Individual consent was obtained from all household members. Medical history, current symptoms, and COVID vaccination status were documented and body temperature was recorded using an infra-red no-touch thermometer. A nasopharyngeal swab (Sigma Virocult®) was taken from all consenting household members by a trained healthcare professional wearing appropriate personal-protective equipment, stored in viral transport medium, and transported to the Mahosot Hospital laboratory in a cool box. The field teams visited the households again and nasopharyngeal swab was repeated for all available cases and contacts on day 7 and day 14 after hospital discharge of each case. Data were entered in a Microsoft ACCESS database.

### Laboratory methods

CILM: Probe-based real-time RT-PCR targeting ORF 1ab and N genes (SanSure Biotech) was performed.

LOMWRU: Patient nasopharyngeal swab samples were extracted using EZ1 Virus mini kit (Qiagen) then submitted to SARS-CoV-2 E-gene qRT-PCR.^12^

NCLE interpretation criteria were used (Ct ≥ 41 = neg; 38≤Ct< 41 = low pos; Ct <38 = pos)

### Ethical approval

The study protocol was approved by the University of Health Sciences Ethics Committee in Laos and the Oxford Tropical Research Ethics Committee (526-21).

## Results

Between 20^th^ May 2021 and 27^th^ August 2021, 55 cases in 27 households and 84 contacts were enrolled in the study. The median [range] age of all 139 included participants was 26.5 years [3 months to 83 years] and 83 (60%) were female. Two cases had received two doses of COVID vaccine. Cases from ten households spent an additional week in a quarantine hotel while others were discharged home from hospital.

Four households did not contribute to the contacts analysis: one because all household members withdrew consent, one because the case went to stay alone somewhere else, and the other two because all household members were cases, leaving 23 households with 67 contacts. No contacts tested positive for SARS-CoV-2 during 14 days of follow up.

### PCR Cycle Threshold (Ct) results for cases

Ct results were not available for all cases at baseline because some were tested in laboratories other than CILM or LOMWRU.

At discharge (day 0) 17 cases had a Ct of 15-34.9, seven had Ct of 35 to <38, and three had Ct of 38-41. On day 7, six had Ct of 35 to <38, four had Ct of 38-41. On day 14, Ct values were 25.2 and 39.99.

## Discussion

Shedding of SARS-CoV-2 RNA may be prolonged for several weeks after the onset of symptoms, however the evidence suggests that in immunocompetent individuals the viral load is too low to transmit around 10 days after infection, regardless of the initial viral load. This is supported by the findings of this pilot study in which patients who still tested positive for SARS-CoV-2 were discharged home and their household contacts were monitored for 2 weeks. No evidence of transmission was demonstrated. Limitations included high rates of withdrawal and loss to follow up (50% by day 14).

## Conclusion

The results of this study support the current policy that it is safe to discharge immunocompetent patients with mild to moderate disease after two weeks’ hospitalisation in Lao PDR, including when PCR results are positive before discharge.

## Figures and Tables

**Table 1:** Participant characteristics

Characteristics	Value (%)
Number of households enrolled	27
Number of cases^1^	55
Number of cases per household; median [range]	1[1-6]
Total number of household contacts	84
Number of contacts per household; median [range]	3[1-13]
Contacts who had received COVID-19 vaccine:	
One dose	32(38%)
Two doses	16(19%)
N cases with positive PCR on D0	32/48(67%)2
N cases with positive PCR on D7	10/47(21%) ^3^
N cases with positive PCR on D14	2/24(8%)
Number of contacts with a positive PCR result during 14 days of follow up ^4^	0
N contacts tested on D0	56/67(84%)
N contacts tested on D7	57/67(85%)
N contacts tested on D14	37/67(55%)

1 A case is anyone who was discharged following a diagnosis of COVID-19, regardless of PCR result on discharge

2 Cases were hospitalized in different centres and not all were tested around the time of discharge

3 Denominator varies as some participants withdrew or were lost to follow up

4 Note contacts denominator reduced to 67 after excluding ineligible households
